# Ley 2287 de 2023 sobre biobancos en Colombia: una reflexión bioética sobre sus alcances

**DOI:** 10.7705/biomedica.7203

**Published:** 2025-03-28

**Authors:** Jorge Mario Vélez-Arango, Doris Yancelly Gil-Espinal, Carolina Hurtado-Montoya, Mónica Massaro-Ceballos

**Affiliations:** 1 Maestría en Bioética, Universidad CES, Medellín, Colombia Universidad CES Universidad CES Medellín Colombia; 2 Grupo de Investigación en Imágenes Médicas, Seguros SURA, Medellín, Colombia Seguros SURA Medellín Colombia; 3 Grupo de Investigación en Enfermedades Cardiovasculares y Pulmonares, Clínica Cardio VID, Medellín, Colombia Clínica Cardio VID Medellín Colombia; 4 Grupo de Ciencias Básicas, Facultad de Medicina, Universidad CES, Medellín, Colombia Universidad CES Universidad CES Medellín Colombia; 5 Grupo Etices, Facultad de Medicina, Universidad CES, Medellín, Colombia Universidad CES Universidad CES Medellín Colombia

**Keywords:** bancos de muestras biológicas, legislación, jurisprudencia, consentimiento informado, bioética, ética en investigación, Biological specimen banks, legislation, jurisprudence, informed consent, bioethics, ethics, research

## Abstract

El creciente interés científico y biotecnológico en los biobancos ha puesto de manifiesto la necesidad de establecer directrices que permitan alcanzar avances importantes de la ciencia y, a su vez, garanticen los derechos de los donantes de muestras biológicas y sus datos asociados. En línea con esta necesidad, el 13 de enero del 2023 se promulgó la Ley 2287 sobre biobancos en Colombia, lo que dio comienzo a un camino que han recorrido otros países desde hace casi 25 años y en el que surgirán nuevos interrogantes que escapan al alcance de esta reglamentación y trascienden como conflictos bioéticos. Este manuscrito pretende resumir aspectos clave de dicha Ley, así como reflexionar sobre sus alcances desde la bioética, en sintonía con la ética de la investigación biomédica y algunas teorías o corrientes bioéticas.

## Abstract (en)

The growing scientific and biotechnological interest in biobanks highlights the need to establish guidelines that allow for important scientific advances and guarantee the rights of the donors of biological samples and their associated data. In line with this need, on January 13, 2023, the Congress of Colombia enacted Law 2287 on biobanks, beginning a path that other countries have followed for almost 25 years. As a result, new questions will arise that are beyond the scope of this regulation and transcend bioethical conflicts. This manuscript aims to summarize key aspects of the mentioned law and reflects on its scope from bioethics, the ethics of biomedical research, and other bioethical currents or theories.

La Ley 2287, por la cual se crea y se regula el Sistema Nacional de Biobancos en Colombia, emitida el 13 de enero de 2023, es de preponderante interés en el contexto de la investigación con muestras biológicas, cada vez más frecuente en biomedicina humana y genética. Con esta nueva ley se pretende regular y establecer el alcance de los biobancos, tema que despierta diversos intereses y retos que requieren explorarse, a nivel nacional e internacional. Según dicha ley, un biobanco con fines de investigación biomédica y biotecnológica se define como «una organización pública o privada, sin ánimo de lucro que posee colecciones de muestras biológicas humanas con datos asociados (personales, clínicos, genéticos y biológicos), bajo parámetros estandarizados y de calidad, cuya finalidad es la de investigar la salud humana» ^1^^,^^2^. En este sentido, la aplicación de la ley propuesta está dirigida a los biobancos constituidos como tales y las instituciones que cuentan con colecciones biomédicas de muestras biológicas y sus datos asociados. 

A continuación, se pretende hacer una revisión de la nueva ley desde su componente técnico-científico y realizar un abordaje bioético sobre las colecciones biomédicas o los biobancos con fines de investigación biomédica y biotecnológica. Para ello, se hizo una búsqueda no sistemática de la literatura en las bases de datos Google académico, Bireme y PubMed, usando el algoritmo:

Search: biobank AND legislation ("biobank s"[All Fields] OR "biobanked"[All Fields] OR "biobankers"[All Fields] OR "biobanking"[All Fields] OR "biological specimen banks"[MeSH Terms] OR ("biological"[All Fields] AND "specimen"[All Fields] AND "banks"[All Fields]) OR "biological specimen banks"[All Fields] OR "biobank"[All Fields] OR "biobanks"[All Fields]) AND ("legislation"[Publication Type] OR "legislation as topic"[MeSH Terms] OR "legislation"[All Fields])

Search: biobank AND bioethic* "biobank s"[All Fields] OR "biobanked"[All Fields] OR "biobankers"[All Fields] OR "biobanking"[All Fields] OR "biological specimen banks"[MeSH Terms] OR ("biological"[All Fields] AND "specimen"[All Fields] AND "banks"[All Fields]) OR "biological specimen banks"[All Fields] OR "biobank"[All Fields] OR "biobanks"[All Fields]) AND "bioethic*"[All Fields].

No se utilizaron filtros de tiempo, tipo de artículo o idioma. Se revisaron algunos textos científicos representativos de los temas abordados. La calidad narrativa del manuscrito de revisión se evaluó según el instrumento SANRA ^3^.

## Biobancos: almacenamiento y uso de muestras biológicas

En este apartado se aborda un poco la historia de los biobancos, algunos referentes normativos internacionales y nacionales, y el concepto de muestra biológica y aspectos relacionados.

El concepto de los biobancos ha cobrado mayor relevancia desde la secuenciación del genoma humano y, más recientemente, con el auge de la investigación traslacional que se sitúa entre la práctica asistencial y la investigación, básica y clínica. La preservación de muestras para su estudio futuro va desde el almacenamiento de muestras sólidas en soluciones a base de formol y parafina -precursores de los biobancos actuales-, hasta la ultracongelación para conservar muestras líquidas. El almacenamiento de muestras biológicas aunado a los vertiginosos avances biotecnológicos y bioinformáticos ha abierto posibilidades inimaginables para los biobancos.

En un principio, se establecieron pequeños repositorios de muestras biológicas. Sin embargo, a la fecha, algunos países poseen biobancos que albergan gran cantidad de muestras de donantes con representatividad poblacional; de hecho, el término biobanco apareció hacia 1994 en la literatura biomédica (https://www.ncbi.nlm.nih.gov/mesh/?term=biobanks), haciendo alusión a estos últimos biobancos, y evolucionó *a posteriori* de una escala poblacional a un enfoque que contempla los derechos individuales y el interés público ^4^.

En sus inicios, las regulaciones para los diversos tipos de biobancos se orientaron a las bases de datos genéticos y, luego, a las colecciones de muestras biológicas en los laboratorios de diagnóstico e investigación ^5^. Durante muchos años, el sentido del material biológico humano recolectado en el marco de la práctica asistencial -como un desecho biológico más que como una parte del cuerpo de una persona que requería protección jurídica-, se transformó con la proliferación de los biobancos a partir de inicios del siglo XXI.

Los primeros biobancos se constituyeron en Europa y estaban orientados a investigar enfermedades prevalentes en la población o a estudiar la genética de poblaciones de países como el Reino Unido, Suecia, Estonia, Islandia, Dinamarca, Noruega y Finlandia. En el 2000, Islandia fue el primer país en contar con una legislación específica ^6^. Estados Unidos comenzó este auge hacia el 2001 y, más tarde, lo hicieron algunos países de Iberoamérica; España es el que tiene la legislación más fuerte ^7^.

Aunque en todos los países la jurisprudencia es coherente con las normas relativas a la investigación biomédica con humanos y las leyes de protección de datos personales, es prioritario contar con una legislación propia para los biobancos de Colombia. Por ejemplo, el marco normativo de países desarrollados como Australia ya contempla la protección de los datos relacionados con la información genética ^8^.

A pesar de que no todos los países de Latinoamérica cuentan con leyes para biobancos, desde el 2008 se estableció la Red de Biobancos de Latinoamérica y del Caribe (REBLAC) con el objetivo de lograr la cooperación científica y técnica en investigaciones transnacionales contra el cáncer. Esta red cuenta con la participación de Brasil, Argentina, Chile, Colombia, Costa Rica, Cuba, Puerto Rico, México, Nicaragua, Ecuador, Perú, Bolivia, Venezuela y Uruguay. Ante las iniciativas con potencial comercial y económico que implican la investigación con muestras biológicas humanas, se requieren normas internacionales con poder vinculante, que estén armonizadas para regular el funcionamiento de los biobancos en el contexto de la investigación traslacional globalizada ^6^.

La creación de los primeros biobancos formalmente constituidos fue contemporánea con la promulgación de las directrices de la Declaración Internacional sobre los Datos Genéticos Humanos del 2003 ^9^, las cuales definen una muestra biológica como:

«[...] cualquier muestra o sustancia biológica (por ejemplo: sangre, piel, células óseas o plasma sanguíneo) que albergue ácidos nucleicos y contenga la dotación genética característica de una persona [...]».

El objetivo de dicha declaración era velar por la protección de los derechos humanos, las libertades fundamentales y el respeto de la dignidad en la recolección, el tratamiento, el uso y la conservación de las muestras, atendiendo a los principios de igualdad, justicia, solidaridad y responsabilidad ^9^.

En este punto vale la pena resaltar que, en la investigación biomédica, los intereses y el bienestar de las personas deben prevalecer sobre los derechos e intereses de la sociedad; y, mencionada esta premisa, surge uno de los aspectos sensibles de los biobancos en el cual coexisten los derechos individuales, el interés público y la investigación científica ^4^.

Entre los referentes para investigaciones relacionadas con la salud humana y los biobancos, es pertinente hacer alusión a las Pautas del Consejo de Organizaciones Internacionales de las Ciencias Médicas (CIOMS), específicamente la N° 11 sobre la recolección, almacenamiento y uso de materiales biológicos y datos relacionados ^10^. En este sentido, Colombia ha ampliado su marco jurídico con la Ley 2287 de 2023, cuyo objeto es:

«[...] Crear el Sistema Nacional de Biobancos, regular la constitución, organización y funcionamiento de los biobancos en Colombia con fines de investigación biomédica y tecnológica para la obtención, utilización, procesamiento, almacenamiento, transporte y cesión de muestras biológicas humanas, sus derivados y muestras relacionadas con la salud humana, así como, su información clínica y biológica asociada, con sujeción a la dignidad e identidad humana, diversidad étnica y cultural del país y los derechos fundamentales de las personas [...]» ^1^.

Dado que los biobancos son los lugares donde se almacenan las muestras biológicas, un aspecto crucial es garantizar el correcto almacenamiento y la seguridad de cada muestra. En consecuencia, los biobancos deben contar con manuales de procedimientos claramente definidos que especifiquen los detalles de los sistemas de almacenamiento, los métodos de identificación y registro de las muestras, el manejo adecuado del material biológico y los protocolos para la destrucción del mismo (dado el caso), además de la asignación de roles concretos al personal que está a cargo ^11^.

La protección de las muestras y de los datos asociados es un factor que incide en la garantía efectiva de los derechos de los donantes en términos de confidencialidad, pues la muestra biológica sigue manteniendo su vínculo con el donante, a pesar de las transformaciones que se realicen para su disociación. Tal vínculo puede ser reversible mediante una codificación (con un código numérico, de letras o de barras), ligada a una identificación personal que está almacenada en otro sitio y que permite la operación inversa hacia la identidad del donante; o irreversible, por medio del anonimato de la muestra, en la que la identificación personal es removida, lo que evita establecer -por medios éticos- el nexo entre la muestra y la información asociada con la identidad del sujeto fuente ^9^^,^^12^. La administración adecuada de esta información en los biobancos garantiza la confidencialidad de los datos y minimiza riesgos que podrían incidir social, económica o políticamente ^13^.

Más allá de la observancia de los derechos humanos respecto a las muestras biológicas y, de manera transversal, a todo el proceso, la fundamentación ética y bioética es cardinal. El respeto, la autonomía, el derecho a la información, la beneficencia, la confidencialidad y la custodia, están anclados a la muestra que debe considerarse, no como una parte del sujeto, sino como una representación integral del individuo ^12^.

Lo descrito hace que la gobernanza de los biobancos deba enfrentar cuestiones éticas trascendentales, como la comercialización de las muestras, la libertad e igualdad en la investigación, la distribución de beneficios, y el riesgo de vulneración de la privacidad y de discriminación genética por mal uso de la información, entre otros ^6^. Por esta razón, el material biológico y sus datos relacionados (recopilados y almacenados en registros de salud de las instituciones que llevan a cabo investigaciones biomédicas) deben contar con sistemas de gobernanza para su uso en futuras investigaciones. El almacenamiento a largo plazo de muestras e información de la salud personal plantea desafíos importantes, desde lo legal y ético, asociados con la gestión de derechos, el consentimiento, la confidencialidad y la propiedad del material biológico. Por lo tanto, no sorprende el animado debate, aún vigente, sobre cómo deberían funcionar los biobancos, las restricciones sobre la participación y la estructura de gobernanza que deberían adoptar ^14^.

## Sumario de la Ley 2287 de 2023

La presente ley establece un glosario de los términos relacionados para estandarizar los conceptos en cuanto a biobancos, con fines de investigación biomédica, biotecnológica y epidemiológica. En el artículo tercero de la ley ^1^, se promulgan algunos principios que buscan garantizar la protección de los derechos de los donantes de muestras biológicas y que están alineados con la Constitución Política de Colombia ^15^. Esta última, en sus principios fundamentales, reconoce la primacía de los derechos inalienables de la persona, sin discriminación alguna, y el derecho a la intimidad. La legislación de biobancos promueve la observancia de los principios bioéticos y de los códigos de buena conducta, y enfatiza el respeto de la autonomía del donante que, en conjunto con la beneficencia, son pilares éticos esenciales, sin excluir los principios mínimos de justicia y de no causar daño ^16^^,^^17^.

Las buenas prácticas en el manejo de las muestras y de sus datos clínicos, genéticos y biológicos asociados están respaldadas por la Política de Ética, Bioética e Integridad Científica de Colombia del 2017 ^18^ y la Declaración de Singapur del 2010 para la integridad en la investigación ^19^^,^^20^. Por su parte, los principios de favorabilidad e interés superior (o mejor interés) -que priorizan la interpretación y la decisión que más favorecen la dignidad y la confidencialidad de las personas (*pro homine*, en jerga jurídica garantista)- y la solidaridad y cooperación internacional según las normas de la investigación biomédica, se encuentran alineados con los pactos por un futuro mejor promovidos por la UNESCO y una ética global ^21^.

La Ley 2287 del 2023 define sus ámbitos de aplicación a biobancos, colecciones biomédicas por fuera del contexto de un biobanco, proyectos de investigación que previamente hayan sido aprobados por el Consejo Nacional de Bioética e instituciones que provean o custodien muestras biológicas humanas, derivados o aislamientos relacionados con salud humana y su información asociada, con fines de investigación biomédica, biotecnológica o epidemiológica.

Las directrices de la ley también se aplican para los profesionales que manipulen cualquier tipo de material biológico de origen humano, que tengan acceso a información clínica, genética y biológica para fines investigativos, y a aquellos responsables de los remanentes del material biológico humano procedentes de intervenciones terapéuticas o diagnósticas que, posteriormente, vayan a ser utilizados en investigación.

La ley también considera la investigación con medicamentos en seres humanos cuando, al finalizar el estudio clínico, incorporen a un biobanco las muestras y derivados junto con la información clínica, genética y biológica. Por último, contempla la entrada o salida de muestras biológicas del territorio nacional con fines de investigación biomédica, biotecnológica o epidemiológica ^1^.

En cuanto a los derechos de los sujetos fuente, o donantes, la ley aborda aspectos como el consentimiento informado, que debe incluir información sobre la investigación en la que se va participar, la posibilidad de acceso a los resultados o aceptación de su socialización, y la protección de los datos genéticos, siempre con primacía del interés superior de la persona fuente o donante ^1^.

En sus otros apartados, la Ley 2287 determina aspectos sobre: la constitución, el funcionamiento y la organización de los biobancos; el almacenamiento, el procesamiento, la cesión y el transporte de muestras biológicas, y el tratamiento de la información asociada; la conformación del Sistema Nacional de Biobancos y la Red Nacional de Biobancos; y, en última instancia, las actividades relacionadas con la inspección y la vigilancia ejercidas por la Superintendencia Nacional de Salud y el Instituto Nacional de Vigilancia de Medicamentos y Alimentos (Invima) ^1^. La función de estos organismos gubernamentales debe ser la de promover, autorizar, apoyar y verificar el funcionamiento de la Red de Biobancos de Colombia, así como conocer el inventario de materiales, datos biológicos y de salud, entre otros, disponibles en el país para facilitar la investigación en salud y fomentar el establecimiento de colecciones biomédicas ^1^.

Por último, es menester mencionar algunas dificultades prácticas en la implementación de la ley, entre ellas, la necesaria articulación interinstitucional y el rol otorgado al Consejo Nacional de Bioética.

## Consentimiento informado en el contexto de biobancos: algunas consideraciones

El proceso de consentimiento informado (o asentimiento informado, si corresponde), es necesario cuando se pretenda utilizar una muestra para investigación en salud. El consentimiento será válido cuando, acorde con la capacidad mental del sujeto fuente o donante ^22^, refleje el respeto por su autonomía y explique el objetivo, las características y los fines del otorgamiento de la muestra, los riesgos potenciales y los resultados de la investigación ^1^^,^^12^. De manera excepcional, podrán usarse muestras biológicas para investigación sin previo consentimiento cuando el sujeto fuente haya fallecido, no haya objeción expresa en voluntades anticipadas o haya un concepto favorable por parte del Consejo Nacional de Bioética. Asimismo, cuando una autoridad sanitaria o médico-legal lo requiera, las muestras biológicas y la información relacionada podrán ser utilizadas siempre que prime el interés general, como en casos de brotes, epidemias, emergencias, desastres o eventos de interés en salud pública, o para establecer las causas, mecanismos o maneras de muerte por parte de la autoridad jurídica ^1^.

En 1997, el *Council of Europe* suscribió el Convenio sobre Derechos Humanos y Biomedicina (conocido también como “Convenio de Oviedo”) con el propósito de tener un fundamento ético en materia de biotecnología ^23^; unos años más tarde, se estableció un protocolo específico para investigación biomédica (Estrasburgo, 2005) ^24^. En el contexto de los biobancos, este convenio contiene aspectos científicos, jurídicos y éticos, que abordan temas relacionados con información y consentimiento, seguridad y supervisión, y confidencialidad y derecho a la información, para proteger la dignidad y la identidad de todos los seres humanos.

Hay cinco aspectos clave que se deben tener en cuenta para salvaguardar el principio del respeto por la autonomía en personas que requieren protección especial, aquellos con limitaciones para manifestar su voluntad por no tener capacidad plena para consentir. Así, con miras a garantizar su bienestar sobre aquel de la ciencia y la sociedad, los principios por considerar son:

- respeto a las condiciones generales de cada individuo;
- ausencia de rechazo por parte de la persona implicada;
- obtención de una autorización escrita del representante legal, en caso necesario;
- eficacia del estudio demostrada en otras personas y
- un beneficio directo para la persona en cuestión ^25^.

De acuerdo con el método de obtención de la muestra y su información asociada, la ley define el consentimiento amplio y el específico: en el primero, se autoriza que las muestras y la información puedan ser cedidas a terceros y utilizadas por diferentes investigadores; en el segundo -el específico- se otorga para un proyecto de investigación concreto y el donante autoriza, expresamente, si acepta ser contactado nuevamente para consentir con miras a otro proyecto. En ambos casos, el sujeto fuente puede revocar el consentimiento en cualquier momento, sin ningún perjuicio ni sanción. Dicha revocatoria o desistimiento aplica sobre la muestra biológica remanente que no se hubiese cedido o procesado, efecto que no es extensivo a los resultados de las investigaciones ya desarrolladas, ni a muestras anonimizadas ^1^. Dos aspectos adicionales para destacar son la gratuidad de la muestra e información asociada, y que la actividad de recolección, custodia y cesión de las muestras del biobanco debe ser sin fines de lucro ^1^.

No sin fundamento, existen temores de atentar contra la autonomía de los donantes de muestras biológicas humanas al implementar un consentimiento amplio, especialmente cuando no se explicitan las restricciones de uso; y, en el caso del consentimiento específico, cuando el proceso para que los sujetos de investigación consientan nuevamente para investigaciones futuras, pueda ser costoso e ineficiente. En ambas circunstancias, se resalta la importancia de la aprobación explícita de una modificación o dispensa del consentimiento por un comité de ética en investigación en seres humanos. Según la pauta N°10 del CIOMS, esta aprobación es posible cuando se cumplen estas tres condiciones:

- no sería factible o viable realizar la investigación sin dicha dispensa o modificación,
- la investigación tiene un valor social importante y
- la investigación entraña apenas riesgos mínimos para los participantes ^10^.

Una alternativa, no contemplada en la presente ley, es el consentimiento dinámico, que permite el contacto bidireccional y en tiempo real (mediante herramientas tecnológicas) de los investigadores y miembros del biobanco con los participantes. De esta manera, los participantes puedan consentir o revocar de manera ágil, como lo hace el proyecto EnCoRe (*Ensuring Consent and Revocation*). Esta última modalidad requiere que el equipo investigador esté enviando permanentemente información relevante de los proyectos de investigación específicos y que el participante brinde todos sus datos para ser contactado, bien sea por correo electrónico o por mensajes de texto. Por supuesto, esta alternativa requiere de desarrollos tecnológicos y recursos, lo cual sería una limitación en las poblaciones que carecen de tales condiciones. Así las cosas, el imperativo es salvaguardar el respeto por la autonomía de los donantes, y lo conveniente es tratar de conservar el equilibrio entre el consentimiento específico y el amplio ^26^.

## Una reflexión bioética del alcance de los asuntos relacionados con biobancos

La preservación y la evolución de la especie humana han estado ligadas, e incluso condicionadas, al desarrollo y uso de la técnica, la ciencia y la tecnología ^27^. Con la convicción de una “ciencia con conciencia”, no se debe asumir una mirada reduccionista de los biobancos como lugares neutrales de almacenamiento o repositorios de conocimiento; todo lo contrario, amerita una profunda reflexión filosófica que implique la ética de la responsabilidad y el cuidado, otros principios bioéticos y el biopoder. Una acotación pertinente en este sentido es que las muestras biológicas humanas comparten un mismo estatuto jurídico con otros datos de salud del sujeto fuente, pues constituyen un reflejo integral del cuerpo de una persona que requiere protección jurídica y pueden tener relevancia para la salud de personas relacionadas con el donante ^6^^,^^28^.

El conocimiento -pasión del hombre desde sus orígenes- y los interrogantes a los que responden las ciencias biomédicas deben ser guiados por la moral y la ética, porque cruzan ese punto que llega a la más íntima ontogenia. En esta era de la medicina genómica, personalizada, predictiva o de precisión y la disponibilidad de datos genéticos a gran escala ^29^ (catalogados como especialmente sensibles dentro de los datos relativos a la salud), existe la posibilidad de llegar a lugares inimaginables del conocimiento. Se tienen las herramientas para generar nuevos descubrimientos que mejoren sustancialmente el bienestar humano, siempre y cuando no sean usados para otros fines poco altruistas, propios de una sociedad de consumo ególatra e individualista.

Ese tenso límite de perfeccionamiento de la especie humana -entre la necesidad y el deseo- a la luz de la medicina, interpela conceptos sobre el cuerpo, la dignidad y la libertad de los seres humanos, que pueden poner en peligro la misma existencia de la humanidad ^27^.

En este orden de ideas, y sin asumir una postura tecnofóbica, la responsabilidad social de la ciencia debe estar enmarcada en el reconocimiento de las personas como sujetos de derechos y no como fuentes biológicas. La ciencia debe priorizar el interés de los donantes de muestras por encima del de la ciencia y la sociedad, y anteponer el respeto a la autonomía frente a los intereses públicos.

Para establecer límites al avance desmesurado en investigación, no es suficiente con crear normas, procurar que se cumplan y estandarizar procesos, sino que debe primar el cuidado del otro. Recientemente, el Papa Francisco se pronunció ante una comunidad científica en Alemania, en relación con las cuestiones éticas sobre el “pensamiento híbrido” entre lo biológico y lo artificial, y enfatizó en el ideal de una investigación con humanidad:

«[...] Porque uno es responsable no solo de lo que hace, sino también y sobre todo de lo que no hace, aunque podría hacerlo [...]» ^30^.

Bien dice este aforismo:

«El científico no solo debe preguntarse: ¿puedo hacer esto?, sino, ¿debo hacerlo? A la primera pregunta responde la ciencia; a la segunda, solo responde la ética» ^31^.

El avance de los biobancos desde el ámbito técnico-científico debe ir en consonancia con una filosofía de las ciencias de la vida, para ser corresponsables de las decisiones personales, colectivas y públicas; además, para anticipar y responder por las consecuencias de las decisiones en el tiempo, sin perjuicio de la humanidad ni del ecosistema presente o futuro ^32^.

Muy a propósito, ante la necesidad de actuar con prudencia frente al enorme poder transformador de la tecnociencia, resulta pertinente hacer alusión a la ética de la responsabilidad con el postulado de Hans Jonas:

«[...] Obra de tal modo que los efectos de tu acción sean compatibles con la permanencia de una vida humana auténtica en la Tierra [...]» ^33^.

La bioética en esta nueva era del bios, entendida como el período de la bioinformática, la megadiversidad y la biología computacional, tiene un desafío importante: educar a buenos ciudadanos y buenos profesionales, responsables de los medios y las consecuencias de sus acciones, y capaces de utilizar las herramientas tecnológicas al servicio de buenos fines ^34^. Esa pretensión cuidadora en el nacimiento de la bioética puede expresarse como:

«”[...] Cuidar lo humano en su vulnerabilidad y en su complejidad [...]”. “[...] Si la bioética se define como una ética de la vida, y la vida es de principio a fin narración, la bioética no tiene más remedio que contar [...]”. “[...] Solo desde el relato tiene sentido una tarea de cuidado. ¿Cómo vamos a cuidar si no conocemos lo que hay que cuidar? [...]”. “[...] ‘Cuidado’ es otra forma de decir ‘responsabilidad’. Cuidar es atender, acoger, procurar, preocuparse, mirar hacia aquello que requiere nuestra atención [...]”. “[...] Somos historias y hemos de cuidar cómo nos contamos, qué relato hacemos de nosotros mismos [...]”» ^27^.

El cuidado de lo humano es esencial en el quehacer bioético y, dado que los seres humanos somos seres de sentido, nuestra identidad es narrativa ^27^. Es aquí donde radica la importancia de un dato personal con su muestra biológica asociada, pues tienen una identidad propia, dotada de sentido. Por ello, la experiencia del cuidar atañe tanto a donadores (sujeto fuente) como a receptores (biobancos).

De nuevo, emerge el respeto por la autonomía y la obligación de salvaguardar la intimidad, la privacidad y la confidencialidad que asiste a unos y otros. Conviene, entonces, revisar con detenimiento estos conceptos que con frecuencia se referencian indistintamente:

«[...] Lo íntimo como lo más interior y reservado (como pensamientos, sentimientos, deseos, creencias, datos genéticos, datos de salud, entre otros); lo privado, que incluiría lo íntimo más todo aquello de carácter personal que sin ser íntimo, no se quiere hacer público; y lo confidencial, como el derecho que asiste al sujeto cuyos datos privados son manifestados en un entorno de confianza, donde quienes reciben dicha información (los ‘confidentes’) no la comunican a terceros, a menos que el propio sujeto lo autorice [...]» ^35^^,^^36^.

Reafirmemos el concepto de cuidado, a la luz de los alcances y potenciales de un biobanco: cuidado es responsabilidad consigo mismo y con la otredad. No en vano Seneca decía: «el hombre es lo más importante para el hombre» y sumado a esa frase culturalmente célebre, viene la de «trata al otro como quieras ser tratado», donde parte la premisa del cuidado de sí. Cuando se tiene la capacidad de autocuidado, se puede pensar en extrapolar ese cuidado al otro, respetando su autonomía y sus libertades, siendo empáticos y compasivos, y buscando tejer relaciones sociales fuertes que se enriquezcan con la diversidad. Es así y solo así que el hombre podrá sobrevivir a sí mismo y a su especie ^37^.

De manera similar, en su obra, Michel Foucault revela una mirada profunda, de afuera hacia adentro, basado en el principio de la filosofía griega “conócete a ti mismo”. El cuidado de sí no es solo conocerse mejor, sino cuidar de sí y de los demás ^38^. Esta reflexión versa sobre la imperiosa necesidad de resignificar el cuidado, esencial desde la bioética para promover una conciencia crítica, ya que el cuidado está en la raíz primigenia del ser humano, es un modo de ser esencial y una dimensión ontológica.

La ética del cuidado implica aplicar principios y valores, universalmente construidos y localmente adoptados, en pro de la dignidad humana. Los derechos humanos y las libertades fundamentales son esenciales para el desarrollo humano integral que implica el fomento de las capacidades individuales y el despliegue pleno de los potenciales de cada persona. Esto facilita el cumplimiento armónico del proyecto vital propio y de los demás congéneres, nutrido por la responsabilidad, la solidaridad y la compasión ^7^^,^^39^.

Día tras día, con más auge, se espera que los biobancos sean plataformas eficaces para la investigación de la biología humana y que respondan cuestiones fundamentales de la vida humana. Hay una creciente disposición de muestras biológicas con datos asociados (epidemiológicos, clínicos, genéticos, ambientales, de hábitos del donante), que representan distintos constituyentes de la función vital y que son aptas para estudios posteriores. Este hecho ha promovido la sistematización e institucionalización de la producción y administración de información biológica humana, con el nivel de detalle que proporcionan las tecnologías informáticas y las “ciencias ómicas”. Con estas últimas, la investigación científica busca garantizar la viabilidad de su propio desarrollo experimental. Sin embargo, los eventuales usos o abusos podrían afectar las prácticas científicas si se arriesga la credibilidad institucional por trasgresiones a la dignidad, debido al manejo descuidado de los datos o por conflictos de interés del material bajo custodia y reserva, entre otros ^28^. Aunque las personas aceptan consciente y voluntariamente la donación de partes de sus cuerpos para apoyar la investigación científica, es deber de los encargados administrar racional y eficientemente estos recursos, dentro de un marco ético y legal óptimo ^28^.

La administración de la información almacenada en los biobancos implica grandes desafíos por el potencial desarrollo de una “biopolítica” en términos de Foucault. En esta época de totalización informacional es imperativo adoptar las debidas precauciones para resguardar la vida y la intimidad de las personas. El poder generado por estos conocimientos, obtenidos a partir de la vida orgánica y social de los sujetos, amerita una sociedad de la vigilancia, una sociedad panóptica, como también diría Foucault ^28^.

Además de los aspectos técnicos inherentes a la recolección, el transporte, la conservación, la identificación o la trazabilidad de las muestras y los datos asociados, los puntos críticos se centran en la gobernanza de los biobancos. Dicha gobernanza está relacionada con la legitimidad de la obtención de las muestras (en los casos en los que se usen para fines no contemplados en el momento de la captura), el resguardo de la intimidad o la privacidad de los individuos fuente, la discriminación por el mal uso de la información asociada (clínica, biológica, genética, étnica, cultural o de cualquier índole), la libertad y la equidad en la investigación, y la distribución de beneficios, entre otros. Lo anterior pone de manifiesto asuntos sumamente sensibles en esta naciente rama del conocimiento en Colombia ^6^^,^^28^.

Si bien una de las pretensiones que se persigue con los biobancos es su alcance trasnacional, cabe mencionar la brecha 10/90 ^40^, sobre la desigualdad en los esfuerzos y recursos destinados a la investigación biomédica entre los países industrializados y los países en desarrollo. Con esto en mente, la destinación de rubros para los biobancos deberá orientarse hacia las prioridades de las comunidades que los albergan y que son la fuente de sus muestras. Aquí entran en escena los comités de ética en investigación como actores fundamentales para la evaluación de la eticidad ^41^ de los proyectos de investigación y como enlaces en la construcción de vínculos de investigación fundados en la confianza y la transparencia, su respeto por los principios éticos, el valor social, la validez científica, la proporción favorable riesgo-beneficio y, en especial, para países como Colombia, la asociación colaborativa con las comunidades de interés ^40^^,^^41^.

En las últimas décadas se han sentado las bases para los biobancos, mediante la estandarización de los procesos brindando orientación y recomendaciones por medio de organismos que dicten las normas y la regulación para la vigilancia, el seguimiento y el control en el cumplimiento y gobernanza local, regional, nacional e internacional. Con estos estándares se busca lograr calidad y validez de los resultados científicos y, ante todo, la protección, el bienestar y la seguridad de los sujetos.

En estas “bibliotecas” de muestras biológicas humanas con fines de investigación, se conjugan lo individual -con su condición humana misma- y la sociedad. Por ello, el lugar de la bioética en un mundo que trae cada vez más innovación y descubrimientos en todos los campos del conocimiento, es promover el diálogo reflexivo entre el *poder hacer* de la biotecnología y el *deber hacer* por responsabilidad social. Se trata de un dilema orientado a la acción y a la toma de decisiones desde el respeto, la dignidad, los derechos y las libertades fundamentales de las personas.

Los autores animan a la construcción teleológica del concepto de biobancos en torno al progreso de las ciencias de la vida, despojándola de la connotación transaccional (banco) e instrumentalizada (cosificación del sujeto) del cientifismo sin alma. En definitiva, no hay reflexiones definitivas en torno al alcance de los asuntos relacionados con los biobancos. Se trata de reconocer la ineludible y bondadosa interacción -aunque no exenta de tensiones- entre el bien individual y el común. En el marco de una ciencia rigurosa con un núcleo de valores humanistas, se persiguen cinco aspiraciones en los fines de la medicina:

«[...] Una medicina... honorable, al frente de su propia vida profesional; moderada y prudente; asequible y sostenible; socialmente sensible y pluralista; y, justa y equitativa [...]» ^42^.

Se espera una pronta reglamentación de la ley a partir de la fecha de su promulgación en enero de 2023, pues es una deuda que tiene el estado colombiano con la comunidad científica y la población general. En consonancia, se proponen tres recomendaciones para su implementación:

- realizar programas de capacitación y sensibilización dirigidos a los profesionales de la salud, investigadores, académicos y demás personal técnico o administrativo involucrado en la gestión de biobancos; estos programas pueden ser ofrecidos a las instituciones de educación superior y a las instituciones prestadoras de servicios de salud, con los propósitos de garantizar que se apropien de los principios éticos y legales establecidos en la ley, y de promover una cultura de respeto y responsabilidad;
- garantizar un sistema de monitoreo y evaluación que permita supervisar las prácticas de los biobancos y el cumplimiento de su funcionamiento ceñido a la ley; esto garantizaría la transparencia y la rendición de cuentas con indicadores precisos y mecanismos de retroalimentación que faciliten la proposición de acciones de mejora y la adaptación de políticas según sea necesario, y
- fomentar el compromiso colaborativo y la participación de las comunidades, los comités de ética y otros actores involucrados en la toma de decisiones relacionadas con la gestión de biobancos. La creación de espacios de diálogo para expresar las preocupaciones y expectativas de cada actor promueven la conciliación de los saberes con las prácticas científicas para un marco de referencia más inclusivo y ético de los biobancos.

A propósito de la demanda social de la ética mínima (civil o pública) ^16^, la filósofa española Adela Cortina expresa: “[...] lo que es necesario, es posible y tiene que hacerse real [...], “[...] porque el mundo que tenemos no está a la altura de lo que merecen los seres humanos [...]” ^43^. La construcción de una ética para una sociedad plural se refleja en el alcance de los biobancos, con su doble dimensión privada (del individuo) y pública (de la sociedad). Este proceso necesita imperativamente del discernimiento moral, ético y jurídico de quienes ejercen la ciencia, la tecnología y la innovación de hoy, para cimentar un futuro más prometedor.
